# Extrahepatic cytochrome P450s play an insignificant role in triptolide-induced toxicity

**DOI:** 10.1186/s13020-018-0179-8

**Published:** 2018-04-23

**Authors:** Yuan Wei, Dujun Wang, Meng Chen, Zhen Ouyang, Shuo Wang, Jun Gu

**Affiliations:** 1 School of Pharmacy, Jiangsu University, 301 Xuefu Road, Zhenjiang, Jiangsu People’s Republic of China; 2 Department of Molecular, Cellular and Biomedical Sciences, University of New Hampshire, Durham, NH USA; 3 Wadsworth Center, New York State Department of Health, 1400 Washington Ave, Albany, NY USA; 4 School of Public Health, State University of New York at Albany, Albany, NY USA

**Keywords:** Triptolide, Xh-CL mouse model, Extrahepatic toxicity, Cytochrome P450, Pharmacokinetic

## Abstract

**Background:**

Triptolide, an active ingredient of Chinese medicine plant *Tripterygium wilfordii* Hook.f., has been shown to exert anti-tumor, immunosuppressive, anti-inflammatory, and anti-fertility pharmacological effects. However, triptolide also causes severe side effects, which are manifested as toxicities in multiple organs. The aim of this study was to analyze the role of extrahepatic cytochrome P450 enzymes in triptolide-induced toxicity.

**Methods:**

Xh-CL mouse model with normal liver, but low extrahepatic P450 expression levels was used in this study. Xh-CL mice and C57BL/6 (wildtype, WT) mice were treated with 200 μg/kg triptolide intraperitoneally every other day for 30 days. The serum levels of alanine aminotransferase (ALT), aspartate transaminase (AST), creatine (Cre), and blood urea nitrogen (BUN) were detected by kits. The changes of tissue were observed with H&E staining. Two groups of mice (Xh-CL and WT animals), were received a single dose of 1 mg/kg TP by oral gavage for pharmacokinetic analysis.

**Results:**

Xh-CL mice displayed higher serum levels of ALT, AST, Cre, and BUN compared to untreated Xh-CL mice. The organ-to-body weight ratio for spleen was high, while that for testes was low. Histopathological changes were observed in multiple organs. However, compared with triptolide-treated WT mice, no significant differences in either blood chemistry or histopathology were recorded. Furthermore, pharmacokinetic studies showed no significant differences between triptolide-treated Xh-CL and WT mice.

**Conclusions:**

Our findings suggest that sub-chronic triptolide treatment can induce toxicities in mouse kidney, spleen, and testis with or without normal local P450 functions. Therefore, extrahepatic P450s play an insignificant role in triptolide-induced toxicity.

**Electronic supplementary material:**

The online version of this article (10.1186/s13020-018-0179-8) contains supplementary material, which is available to authorized users.

## Background

Triptolide (TP) is the primary active diterpenoid triepoxide isolated from *Tripterygium wilfordii* Hook.f. (“thunder god vine” in Chinese herbal medicine). TP exhibits anti-inflammatory, immunosuppressive, anti-fertility, and anti-tumor effects [1, 2]. TP has also shown potential in the treatment of autosomal dominant polycystic kidney disease [3]. However, clinical use of TP is limited because of its toxic effects and narrow therapeutic window. The side effects of TP include gastrointestinal discomfort, amenorrhea, kidney dysfunction, leukopenia, thrombocytopenia, and aplastic anemia, and they have been reported to occur in more than 45% of patients [4].

Previous studies have confirmed that cytochrome P450s are responsible for the hydroxylation of TP in vitro [5], and higher expression of hepatic CYP3A can facilitate the biotransformation of TP and decrease its hepatotoxicity in rats [6, 7]. These findings suggested that the decreased activity of hepatic CYP3A was a key factor responsible for the adverse effects associated with TP in vivo. A clinical study assessing 202 Chinese individuals showed that the hepatic CYP3A metabolic activities were normally distributed [8]. In other words, significantly low hepatic CYP3A metabolic activity was found only in a small percentage of the studied group, indicating that low activity of hepatic CYP3A, as a single factor, was not sufficient to explain why nearly half of patients treated with TP experienced adverse reactions [4]. Therefore, other related factors affecting TP-induced toxicity should be identified. A previous report showed that most of the TP-induced adverse reactions occurred in extrahepatic tissues [6]. We therefore focused on the role of extrahepatic P450 activities.

A recent study with a liver-specific cytochrome P450 reductase knockout mouse model (LCN mouse) [9] confirmed that inactivation of hepatic P450s abolishes metabolism of TP in the liver, resulting in an increase in TP bioavailability and toxicity in vivo. Xue et al. found that while the role of hepatic P450s in TP-induced toxicity was well defined, the function of extrahepatic P450s could not be assessed because TP circulation levels were greatly increased in the LCN mouse model. This problem may be overcome by using a mouse model that expresses only low levels of extrahepatic cytochrome P450 reductase (Xh-CL). The Xh-CL mouse model was generated by crossing a reversible-CL (r-CL) mouse model with the albumin-Cre mouse model. Xh-CL mice show normal cytochrome P450 reductase (CPR) expression in hepatocytes, while the expression levels in other tissues including brain, kidney, lung, OM, testis, and ovary were only 4–24% of the corresponding levels found in wildtype (WT) mice. No significant differences in liver microsomal activities were found between Xh-CL and WT mice [10]. In this study, we treated Xh-CL mice and WT (C57BL/6) mice with TP and compared toxicity and pharmacokinetics.

## Methods

### Materials and chemicals

TP (> 99% purity) was purchased from ChromaDex (Irvine, CA, USA). It was dissolved in Tween80 (Sigma-Aldrich, St. Louis, MO, USA), and then diluted to the required concentration in 0.9% saline, for a final Tween 80 concentration of less than 1% v/v. Alanine aminotransferase (ALT), aspartate transaminase (AST), blood urea nitrogen (BUN), and creatine analysis kits were purchased from Nanjing Jiancheng Bioengineering Institute (Nanjing, China).

### Experimental animals and drug administration

Cpr-low (CL) mice were kindly provided by Prof. Xinxin Ding (Wadsworth Center, Albany, NY, USA). Alb-Cre and C57BL/6 mice were purchased from the Model Animal Research Center of Nanjing University (Nanjing, China), permit number SKXK (Su) 2010-0001. Xh-CL mice were produced by the crossing of the Cpr-low (CL) and Alb-Cre mice as reported previously [10]. The animals were provided with a standard laboratory diet and tap water ad libitum during the experiments. 8-week-old male WT mice weighing 20 ± 2 g were used for experiments. In the general toxicological studies, Xh-CL and WT mice (n = 5) were administered 0.1 and 0.2 mg/kg TP by i.p. injection, and the control groups received a vehicle [11]. The mice were dosed at approximately 9:00–10:00A.M. every other day for 30 days. For the pharmacokinetic study, the animals were dosed with 1 mg/kg TP by oral gavage. All animal experiments were approved by the Institutional Animal Ethics and Use Committee of Jiangsu University. The minimum standards of reporting checklist contains details of the experimental design, and statistics, and resources used in this study (Additional file 1).

### General characterization of TP-induced toxicity

The mice were weighed and euthanized with carbon dioxide 24 h after the last TP treatment. Blood was collected by cardiac puncture. Serum samples were prepared by centrifugation after coagulation for 30 min at room temperature. Serum levels of ALT, AST, BUN, and creatinine were determined using commercial kits. Organs (brain, liver, lung, kidney, spleen, and testes) were weighed at the time of necropsy. The organ: body weight ratios were calculated. Next, partial tissues were fixed in 10% neutral buffered formalin for histological examination, remaining tissues were frozen at − 80 °C. The tissue sections (5-µm thickness) were stained with hematoxylin and eosin (H&E) for pathological analysis, which was carried out by Prof. Miao Chen of the Department of Pathology, First People’s Hospital, Zhenjiang, China. For semi-quantitative assessment of the extent of tissue toxicity, the severity of tissue lesions was graded as follows: +, moderate; ±, mild; and −, negative [12].

To determine the tissue distribution of TP in mice, frozen tissues were homogenized in saline (1.0 g wet weight/mL) on ice. Triptolide was extracted from the tissue homogenates including liver, kidney, spleen and testes. Each sample was extracted with an equal volume of ethyl acetate for three times, and then dried under nitrogen. The residues were reconstituted in 50 µL methanol for analysis.

TP concentrations were quantified with an Agilent 1260 Infinity Liquid Chromatography system coupled to the Thermo LXQ™ Linear Ion Trap mass spectrometer (Thermo Fisher Scientific, Waltham, MA, USA). The mobile phase consisted of acetonitrile and water (30:70), with a flow rate of 0.2 mL/min. An electrospray interface in negative ionization mode was used. ESI source parameters were as followed: sheath gas flow rate 10.5 L/min, capillary temperature 325 °C, capillary voltage 3500 V, nebulizer pressure 25 psi, quality scan range (*m/z* 100–1200, fragmentor 110 eV, collision energy 70 eV). Xcalibur™ software (Thermo Finnigan) version 1.2 was used to analysis the data (Thermo Fisher Scientific, Waltham, MA, USA).

### Pharmacokinetic analysis

Two groups of mice (Xh-CL and WT animals, 24 mice in each group) were used for pharmacokinetic analysis. The mice received a single dose of 1 mg/kg TP by oral gavage. Blood was collected at 5, 10, 15, 20, 30, 45, 60, and 120 min (n = 3 for each time point). Around 0.5 mL blood was collected from the ocular sinus at each time point. The mice were euthanized with CO_2_ immediately after blood collection. Plasma was prepared by centrifugation at 900×*g* for 10 min and kept at − 80 °C until analysis. TP was then extracted from 200 µL plasma with 2 × 600 µL ethyl acetate and dried under nitrogen. The residues were reconstituted in 100 µL methanol for analysis. TP concentrations were quantified with an Agilent 1290 Infinity Liquid Chromatography system equipped with Agilent ZORBAX Eclipse Plus C18 column (2.1 × 100 mm, 1.8 μm) (Agilent Technologies, Santa Clara, CA, USA) for pharmacokinetic analysis. The mobile phase consisted of acetonitrile and water (30:70), with a flow rate of 0.2 mL/min and a sample volume of 2 µL. The compound was measured at 218 nm, and the column was maintained at 25 °C. The pharmacokinetic parameters were analyzed with the software package DAS 3.2 (Mathematical Pharmacology Professional Committee of China, Shanghai, China).

### Statistical analysis

All data are expressed as mean ± standard deviation (SD). Data were evaluated for statistical significance by one-way analysis of variance (ANOVA) or Student’s *t* test. SPSS 13.0 (IBM SPSS, Armonk, NY, USA) was used for analysis, and *p* < 0.05 was considered statistically significant.

## Results

### Blood chemistry parameters

**Fig. 1:**
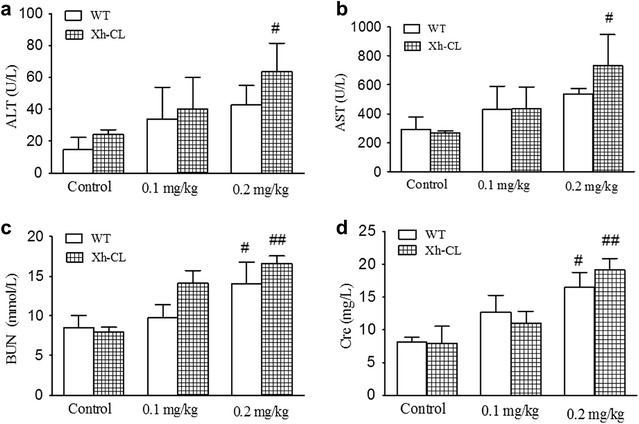
Effects of triptolide (TP) intraperitoneal (i.p.) injection on serum biochemical parameters in Xh-CL mice and WT mice. TP was administered at doses of 0.1 and 0.2 mg/kg; the control mice were administered with a vehicle, mice were dosed every other day for 30 days. **a** Alanine aminotransferase (ALT); **b** aspartate transaminase (AST); **c** creatinine; **d** blood urea nitrogen (BUN). ^#^*p* < 0.05, ^##^*p* < 0.01 vs. control

We first measured AST and ALT levels to assess mouse liver function, and creatinine and BUN levels to evaluate renal function in each group. As shown in Fig. 1a, b, the serum ALT and AST levels in Xh-CL mice treated with 0.2 mg/kg TP differed from those in control Xh-CL mice, indicating liver cell damage or abnormal function (likely acute toxic hepatitis). However, Xh-CL mice treated with 0.1 mg/kg TP showed no significant differences in the levels of kidney and liver toxicity markers compared with the control Xh-CL mice (Fig. 1a, b). Similarly, the serum creatinine and BUN levels in Xh-CL mice treated with 0.2 mg/kg TP differed from those in the control (Fig. 1c, d), indicating renal cell damage or abnormal renal function, and the mice may possibly be suffering from glomerulonephritis. Notably, no significant differences were observed in AST, ALT, creatinine, and BUN levels between Xh-CL mice and WT mice (Fig. 1c, d).

### Organ: body weight ratios and histopathology analysis

To assess the sub-chronic toxicity of TP, we compared the organ: body weight ratio of various organs between Xh-CL and WT mice. Table 1 shows that Xh-CL and WT mice treated with 0.1 mg/kg TP exhibited no significant differences in organ-to-body weight ratios for any organ compared with the controls. However, significantly increased spleen to body weight ratio was observed in Xh-CL mice treated with 0.2 mg/kg TP (*p* < 0.05) concomitantly with significantly decreased testis weight (*p* < 0.01). In WT mice treated with 0.2 mg/kg TP, liver to body weight ratios were significantly higher (*p* < 0.05), while testis to body weight ratios were significantly lower (*p* < 0.05) when compared with the control mice.

**Table 1:** Effects of triptolide on organ: body weight ratios

Organ	Mouse group	Organ: body weight ratios (%)		
		Control	0.1 mg/kg	0.2 mg/kg
Brain	Xh-CL	2.01 ± 0.13	2.17 ± 0.19	1.98 ± 0.01
	WT	2.45 ± 0.13	2.44 ± 0.02	2.32 ± 0.06
Liver	Xh-CL	5.67 ± 0.53	5.43 ± 0.13	6.62 ± 0.80
	WT	4.94 ± 0.86	4.54 ± 0.79	7.01 ± 0.25^#^
Lung	Xh-CL	0.64 ± 0.03	0.73 ± 0.05	0.63 ± 0.01
	WT	0.65 ± 0.01	0.66 ± 0.01	0.64 ± 0.02
Kidney	Xh-CL	1.41 ± 0.06	1.52 ± 0.45	1.54 ± 0.03
	WT	1.40 ± 0.01	1.54 ± 0.08	1.63 ± 0.01
Spleen	Xh-CL	0.34 ± 0.01	0.42 ± 0.02	0.59 ± 0.04^#^
	WT	0.47 ± 0.02	0.34 ± 0.01	0.61 ± 0.01
Testis	Xh-CL	0.87 ± 0.11	0.63 ± 0.01	0.40 ± 0.03^##^
	WT	0.74 ± 0.04	0.57 ± 0.03	0.43 ± 0.06^#^

Xh-CL and WT mice (n = 5 in each group) were administered with 0.1 or 0.2 mg/kg triptolide. The control mice were administered with a vehicle. Mice were dosed every other day for 30 days. Data were shown as mean ± SD

^#^*p* < 0.05, ^##^ *p* < 0.01 vs. control

**Fig. 2:**
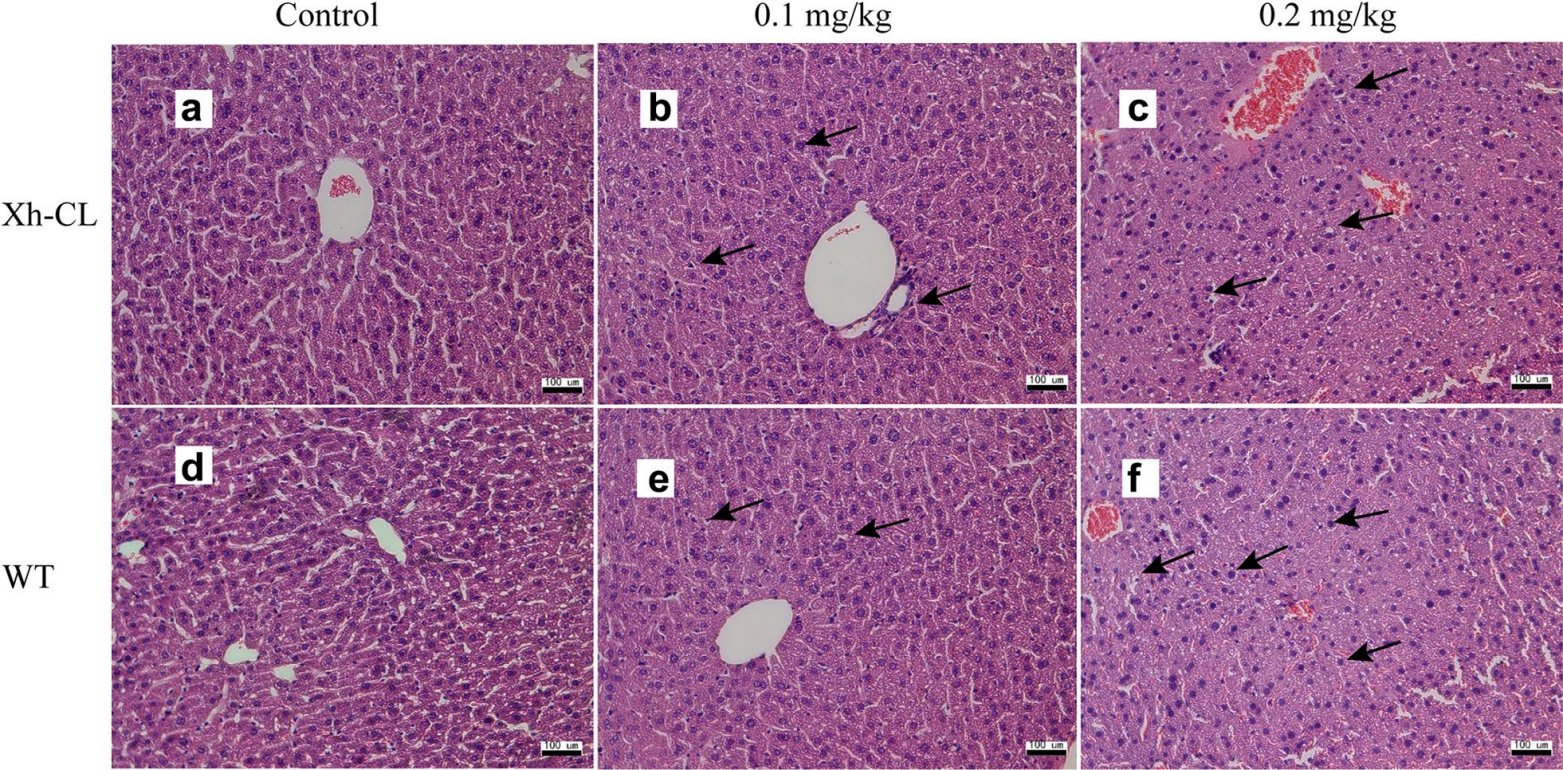
Comparison of liver lesions in Xh-CL and WT mice following triptolide oral treatment (H&E staining, ×200). The mice were administered with 0.1 mg/kg (**b**, **e**) or 0.2 mg/kg (**c**, **f**) triptolide. The control mice received a vehicle (**a**, **d**). Mice were dosed every other day for 30 days. Arrows indicate necrotic and swollen hepatocytes

**Fig. 3:**
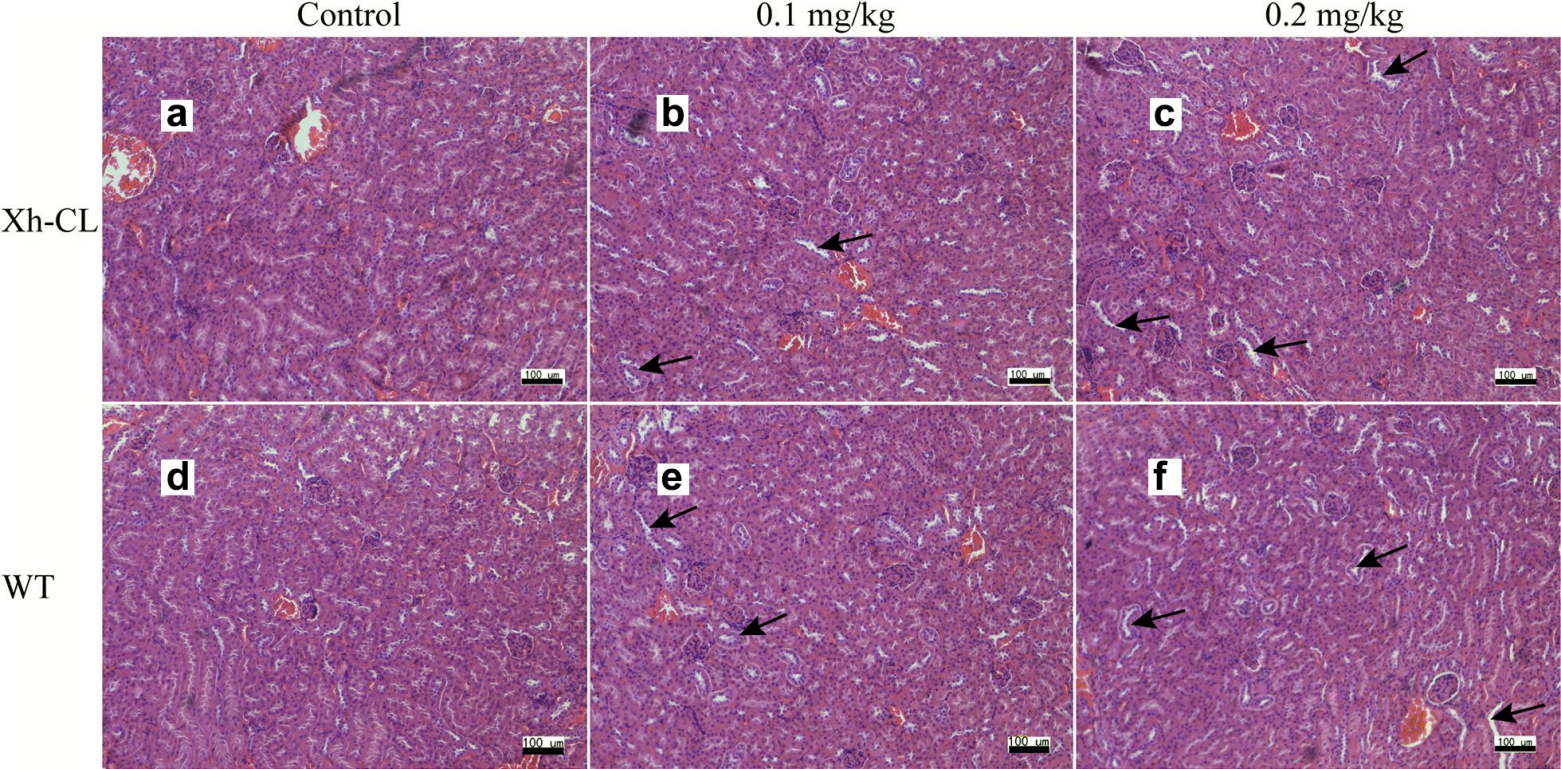
Comparison of kidney lesions in Xh-CL and WT mice following triptolide oral treatment (H&E staining, ×200). The mice were administered with 0.1 mg/kg (**b**, **e**) or 0.2 mg/kg (**c**, **f**) triptolide. The control mice received a vehicle (**a**, **d**). Mice were dosed every other day for 30 days. Arrows indicate renal proximal tubular dilation; some protein casts are visible

**Fig. 4:**
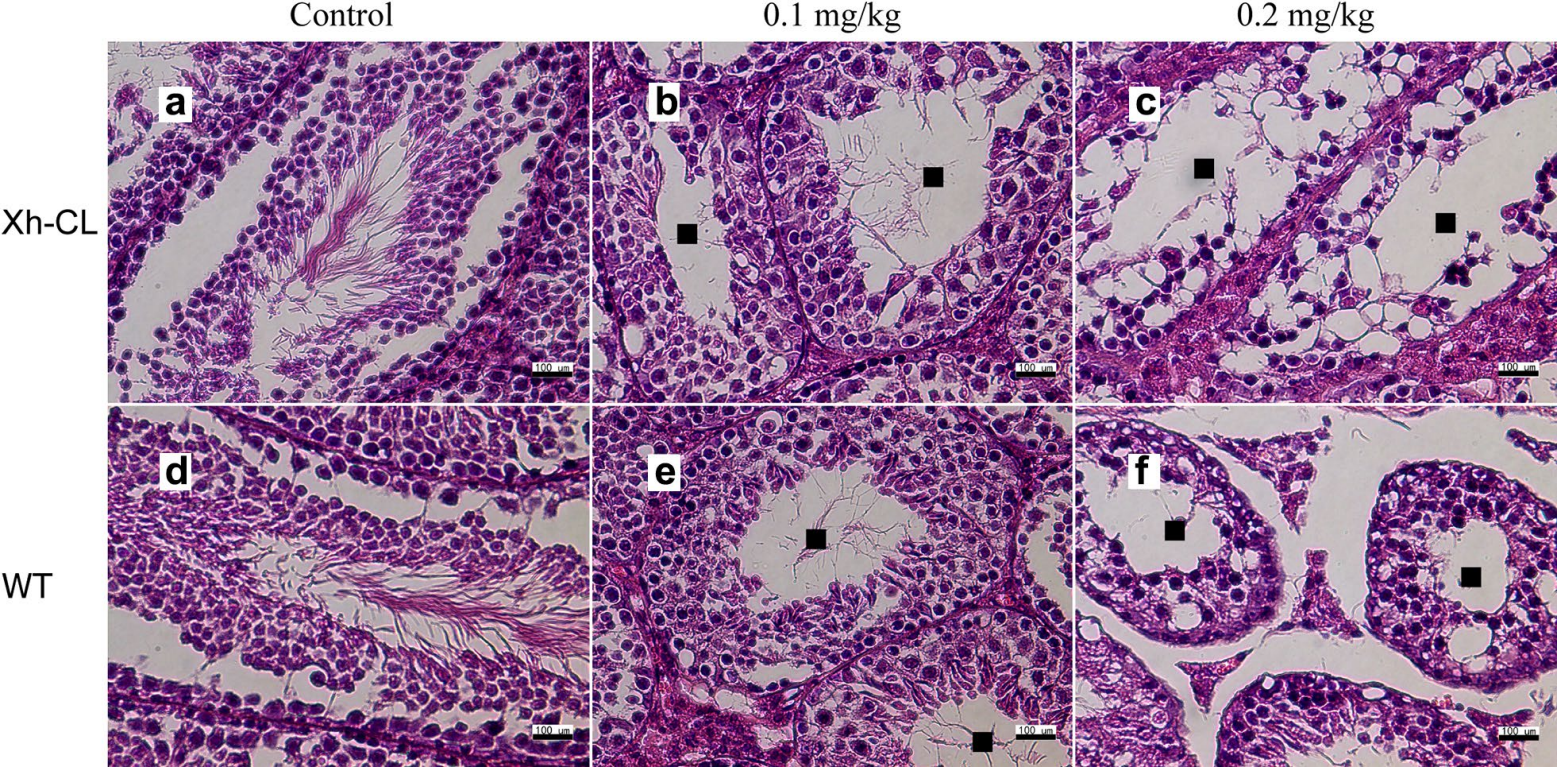
Comparison of testicular lesions in Xh-CL and WT mice following triptolide oral treatment (H&E staining, ×200). The mice were administered with 0.1 mg/kg (**b**, **e**) or 0.2 mg/kg (**c**, **f**) triptolide. The control mice received a vehicle (**a**, **d**). Mice were dosed every other day for 30 days. Squares indicate reduced primary and secondary spermatocytes in the seminiferous tubules

**Fig. 5:**
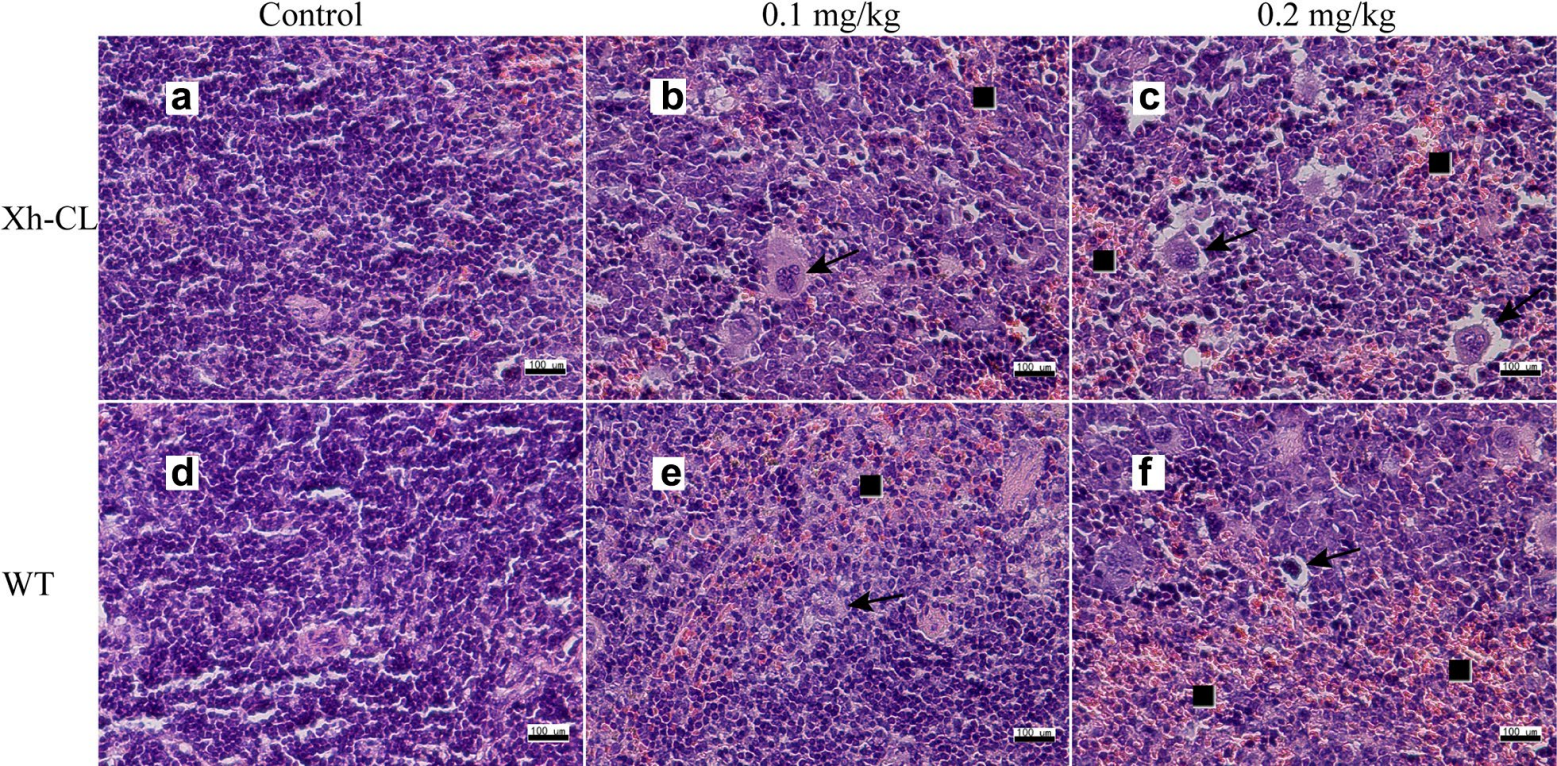
Comparison of splenic lesions in Xh-CL and WT mice following triptolide oral treatment (H&E, ×200). The mice were administered with 0.1 mg/kg (**b**, **e**) or 0.2 mg/kg (**c**, **f**) triptolide. The control mice received a vehicle (**a**, **d**). Mice were dosed every other day for 30 days. Squares indicate bleeding of dilated splenic sinus. Arrows indicate splenic atrophy

**Fig. 6:**
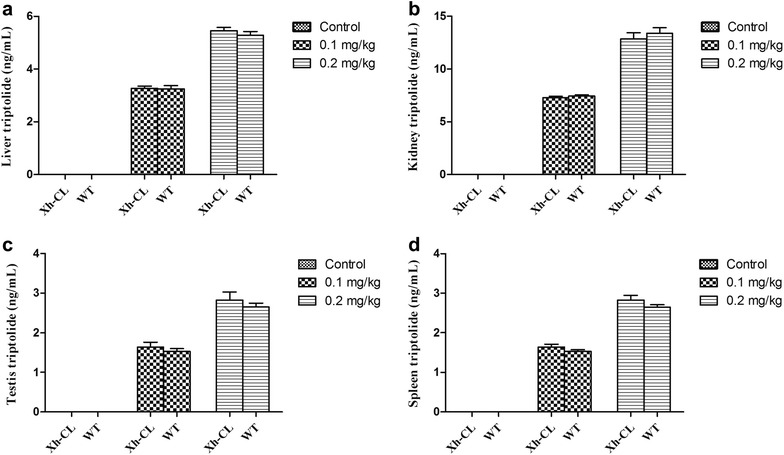
Levels of triptolide in the liver (**a**), kidney (**b**), testis (**c**) and spleen (**d**) of Xh-CL and WT mice 24 h after oral treatment. The mice were administered with 0.1 or 0.2 mg/kg triptolide. The control mice received a vehicle. Mice were dosed every other day for 30 days. Mean ± standard deviation (indicated by error bars) are shown. No significant difference was observed between the groups at each dosage

Figure 2 shows the representative images of hepatic lesions in the different exposure groups. Histological analysis revealed some necrotic and swelling hepatocytes in both Xh-CL and WT mice following treatment with 0.2 mg/kg TP (Fig. 2c, f). The representative images of renal lesions in the different groups are shown in Fig. 3. Kidney proximal tubular epithelial cell dilation and some protein casts were found. Glomerular capillary loop lesions were also found in both Xh-CL and WT mice following 0.2 mg/kg TP treatment (Fig. 3c, f). The representative images of lesions in the testes are shown in Fig. 4. In both groups treated with 0.2 mg/kg TP, primary and secondary spermatocytes were reduced (or completely absent) in the seminiferous tubules. In addition, testicular edema was present (Fig. 4c, f). Figure 5 compares the spleen lesions found in the exposure groups. Splenic sinus dilation and splenic atrophy were observed in both the groups treated with 0.2 mg/kg TP (Fig. 5c, f). These findings suggest that TP induces toxicity in multiple organs in both Xh-CL and WT mice, with similar patterns. The extent of tissue toxicity is shown in Table 2. More severe lesions were found in Xh-CL and WT mice after treatment with 0.2 mg/kg TP. Of the five Xh-CL mice, one exhibited moderate hepatoxicity, while three showed mild liver toxicity, and of the five WT mice, one displayed moderate hepatoxicity, while two showed mild toxicity. Two of the five Xh-CL mice exhibited moderate renal toxicity, while two showed mild toxicity. One of the five WT mice displayed moderate renal toxicity, and two showed mild toxicity. All mice displayed moderate testicular toxicity in both Xh-CL and WT groups. Two of the five Xh-CL mice displayed moderate splenic toxicity, while two showed mild toxicity. One of the five WT mice exhibited moderate splenic toxicity, and three showed mild toxicity. Levels of triptolide in the liver, kidney, testis and spleen of Xh-CL and WT mice were detected, no significant difference was observed between the groups at each dosage (Fig. 6a–d).

**Table 2:** Extent of tissue toxicity in Xh-CL and WT mice following triptolide

Organ	Mouse group	Triptolide dose (mg/kg)	Number of mice in each grade		
			+	±	–
Liver	Xh-CL	0.1	0	2	3
		0.2	1	3	1
	WT	0.1	0	1	4
		0.2	1	2	2
Kidney	Xh-CL	0.1	0	2	3
		0.2	2	2	1
	WT	0.1	0	2	3
		0.2	1	2	2
Testis	Xh-CL	0.1	2	3	0
		0.2	5	0	0
	WT	0.1	1	4	0
		0.2	5	0	0
Spleen	Xh-CL	0.1	0	1	4
		0.2	2	2	1
	WT	0.1	1	1	3
		0.2	1	3	1

Xh-CL and WT mice (n = 5 in each group) were treated with 0.1 or 0.2 mg/kg triptolide, mice were dosed every other day for 30 days. The severity of lesions in the tissues was graded as follows: +, moderate; ±, mild; and −, negative

### Pharmacokinetic analysis

**Fig. 7:**
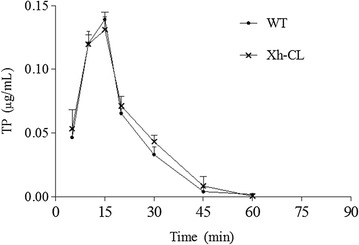
Triptolide (1.0 mg/kg) clearance in Xh-CL and WT mice. Mean ± standard deviation (indicated by error bars) are shown. No significant difference was observed between the groups at any time point

The plasma concentrations of TP were plotted against time curves (Fig. 7). The calculated pharmacokinetic parameters are summarized in Table 3. Plasma TP reached peak levels at 15 ± 0 min in both mouse groups. Other pharmacokinetic parameters, including C_max_, the area under the curve (AUC), T_1/2_, and CLz/F in the Xh-CL mice were remarkably similar to those in WT mice. These findings indicate that TP was cleared from both Xh-CL and WT mice in a similar manner.

**Table 3:** Triptolide plasma pharmacokinetic parameters in Xh-CL and WT mice

Group	C_max_ (mg/L)	T_max_ (min)	AUC_(0–t)_ (mg/L min)	AUC_(0–∞)_ (mg/L min)	T_1/2_ (min)	CLz/F (L/min/kg)
Xh-CL	0.145 ± 0.002	15 ± 0	2.435 ± 0.233	2.445 ± 0.275	6.411 ± 1.41	0.409 ± 0.029
WT	0.139 ± 0.004	15 ± 0	2.579 ± 0.194	2.602 ± 0.198	7.442 ± 1.64	0.384 ± 0.035

Xh-CL and WT mice were administered 1.0 mg/kg triptolide orally. All data are presented as mean ± standard deviation. No significant differences were observed between the groups for any parameter (n = 3). Data were shown as mean ± SD

*C*_*max*_ maximum concentration, *T*_*max*_ time to reach C_max,_ *AUC* area under the curve, *T*_*1/2*_ elimination half-life, *CLz/F* oral clearance

## Discussion

Cytochrome P450 enzymes are involved in the biotransformation of most xenobiotic compounds [13]. The liver is usually regarded as the most important organ in cytochrome P450-mediated drug metabolism, while P450s expressed in various extrahepatic tissues can also contribute to target tissue toxicity induced by tissue-selective toxicants [14].

The mechanism underlying TP-induced toxicity has not yet been elucidated clearly [15]. Theoretically, TP-induced toxicity can be caused either by the parent compound itself or by its reactive metabolites generated through biotransformation reactions. The extrahepatic metabolites can be produced by local P450s or can be generated in the liver or other tissues and then transported to the target tissues by systemic circulation [16]. A study with liver-Cpr-null mouse model reported that inactivation of hepatic P450s suppresses TP metabolism in the liver, leading to an increase in the bioavailability and toxicities of the compound [9]. However, the role of cytochrome P450s in extrahepatic organs was not discussed extensively in the aforementioned study, because extrahepatic toxicity was confounded by the reduced hepatic clearance of TP. This reduction led to greater systemic bioavailability, resulting in possible overestimation of the extrahepatic toxicities. In this study, we evaluated the role of extrahepatic P450 enzymes in triptolide-induced toxicity using Xh-CL mice with normal liver, but low extrahepatic levels of cytochrome P450 enzymes. The knockdown of extrahepatic P450 enzymes in mice could not change either the general toxicities, local tissue levels, or the pharmacokinetics of TP. Thus, we found that extrahepatic P450 was probably not a significant factor in TP-induced toxicity. Results from the Xh-CL model provided direct evidence for the involvement of extrahepatic CPR-dependent enzymes in extrahepatic tissues, without confounding from reduced hepatic metabolism.

The small intestine is involved in first-pass metabolism of orally ingested xenobiotics, especially the substrates of CYP3A [14, 17]. CYP3A is the predominant cytochrome subfamily in the small intestine, accounting for 70–80% of total intestinal cytochrome content in humans [18]. In mice, intestinal CYP3A was first detected by erythromycin and cyclosporine activities and by immunoblot analysis [19]. The expression of CYP3A in the mouse small intestine was further confirmed by a mouse everted gut sac model [20] and by a systemic analysis of P450s expressed in the mouse small intestine and their inducibility [21]. However, in this study, the reduced CYP3A activity in the small intestine of Xh-CL mice appeared to exert few effects on the pharmacokinetics of TP and TP-induced toxicities.

The TP-induced toxicities were often studied with acute exposures in mice, but liver toxicities were hard to detect in wildtype animals without high doses (e.g. 1.0 mg/kg) close to LD_50_ of TP [6, 22]. In our sub-chronic toxicity study, we generated hepatic toxicological profiles for TP in C57BL/6 mice with moderate doses (0.1 or 0.2 mg/kg). High serum AST and ALT levels were detected, and they were indicatives of liver damage due to liver cell necrosis and cytosol leakage into the serum [23]. Our histological analysis also showed necrotic and swollen hepatocytes in the liver tissue sections. Since *Tripterygium wilfordii* Hook.f. was often used for long-term treatment in traditional Chinese medicine practices [24], data from our sub-chronic study could be helpful to get a better understanding of TP-induced clinical toxicity.

## Conclusions

In conclusion, our findings indicate that sub-chronic TP treatment can cause toxic effects in multiple organs in mice, with or without extrahepatic P450 activity. To reduce TP-induced toxicity in clinical settings, related factors other than extrahepatic P450 enzymes should be identified and characterized.

## Additional file

**Additional file 1.** Minimum standards of reporting checklist.

## Abbreviations

ALT: alanine aminotransferase
AST: aspartate transaminase
BUN: blood urea nitrogen
Cre: Cre recombinase
CPR: cytochrome P450 reductase
r-CL: reversible-Cpr low
Xh-CL: extrahepatic-CL
TP: triptolide
WT: wild type
